# Risk factors for short-term postoperative complications in the 8 weeks after tibial plateau leveling osteotomy in dogs weighing less than 15 kilograms: A retrospective study

**DOI:** 10.1371/journal.pone.0247555

**Published:** 2021-02-25

**Authors:** Karen Marin, Marcos D. Unis, Jason E. Horgan, James K. Roush

**Affiliations:** 1 LeadER Animal Specialty Hospital, Cooper City, Florida, United States of America; 2 Department of Clinical Sciences, Kansas State University, Manhattan, Kansas, United States of America; Faculty of Veterinary Medicine - University of Lisbon, PORTUGAL

## Abstract

The purpose of this retrospective study was to evaluate the risk factors for short-term postoperative complications in the 8 weeks after unilateral tibial plateau leveling osteotomy (TPLO) for cranial cruciate ligament rupture in small dogs weighing less than 15 kg. Medical records were retrospectively reviewed for 90 dogs weighing <15 kg that underwent medial parapatellar arthrotomy with inspection of the meniscus and TPLO performed by the same surgeon between January 2012 and December 2017. The overall complication rate was 4.44% (4/90 dogs). There were four cases of partial incisional dehiscence, none of which required surgical revision. Complications were significantly more likely in dogs that had undergone placement of a 2.4-mm TPLO plate. Overall, the complication rate was less than that in previous studies of dogs weighing > 15kg. In this study, patients in which 2.4-mm TPLO plates were used were more likely to develop postoperative complications. Dogs weighing <15 kg that underwent TPLO had good short-term outcomes with minimal complications. In our study, the overall complication rate after TPLO in dogs weighing <15 kg is less than that historically reported in heavier dogs. Our data suggests that TPLO is a safe treatment option in small dogs with cranial cruciate ligament rupture.

## Introduction

The most common orthopedic problem in dogs is rupture of the cranial cruciate ligament (CCL) [1, 2], which serves as a major stifle stabilizer. Several techniques can be used to stabilize the stifle after CCL rupture, the most common of which is tibial plateau leveling osteotomy (TPLO) [2]. TPLO stabilizes the stifle by neutralizing dynamic tibiofemoral shear forces during the stance phase of the gait cycle [3].

In smaller dogs, veterinary surgeons tend to perform an extracapsular repair, such as placement of a lateral fabellar suture, rather than a TPLO [2, 4]. In a survey of American College of Veterinary Surgeons diplomates and primary care veterinarians, size was cited as one of the most important determinants of the procedure used in dogs [2]. The most common treatment recommendation for CCL pathology was extracapsular stabilization in the given scenario of a small dog (weighing 9.1 kg) and TPLO in a large dog (weighing 27.2 kg) in both groups [2].

Many practitioners suggest that small dogs (weighing <15 kg) can be treated conservatively by long-term use of anti-inflammatory agents, pain medication, and restricted activity with a good functional outcome [5]. Nonsurgical management was reported to be the first treatment step in dogs weighing <15 kg by 84% of 161 veterinarians surveyed in the UK [4]. Several studies have found that CCL rupture develops later in life in dogs weighing <15 kg than in larger dogs [6]. Furthermore, one study reported that histologic changes associated with CCL were less severe in dogs weighing up to 15 kg than those in heavier dogs and that the onset of the degenerative process was delayed by several years [7].

Several studies have evaluated the complications after TPLO in large-sized and giant-sized dogs. The TPLO complication rates range from 10% to 34% and vary in severity from swelling and bruising to fractures and osteomyelitis [3, 8–13]. Implant-related complications reportedly occur in <10% of all TPLO procedures [8, 9, 11]. There is also evidence of a significant association between body weight and the overall complication rate after TPLO [13–16]. Surgical site infection rates of 21.3%-25.9% after TPLO have been reported for giant-breed dogs weighing >50 kg [17, 18]. In a recent study, treatment of large dogs with an excessive tibial plateau angle (TPA) was associated with a significantly higher complication rate but with an outcome comparable to that of dogs without an excessive TPA [19].

Risk factors for CCL rupture and complications after TPLO in small-breed dogs have not been extensively studied. The objectives of this retrospective study were to investigate the short-term complications of TPLO surgery with arthrotomy in dogs weighing <15 kg in the 8 weeks after surgery. The purpose was to identify whether or not age, weight, sex, neutering status, TPA, surgical time, plate size, type of closure, or meniscal treatment were risk factors for postoperative complications. We also investigated whether or not the complications in dogs weighing <15 kg were comparable with previously reported complication rates. To our knowledge, this is the largest case study of the short-term complications of TPLO surgery in dogs weighing <15 kg.

## Materials and methods

### Study design

Electronic medical records from dogs that were presented at LeadER Animal Specialty Hospital between January 2012 and December 2017 were de-identified before analysis. The study inclusion criteria were as follows: weight <15 kg, unilateral TPLO performed for CCL rupture, and at least 8 weeks of follow-up that included physical examination and radiographs. CCL rupture was diagnosed preoperatively by physical examination that included the cranial drawer and cranial tibial thrust tests. Dogs with concurrent medial patellar luxation (MPL) were excluded.

The data collected included age, breed, sex, neutering status, weight, preoperative and postoperative TPA, damage and treatment of the meniscus, implant size, surgical time, closure method (use of staples vs sutures), postoperative antibiotics administered and postoperative complications. Orthogonal radiographs of the affected stifle were obtained preoperatively, immediately postoperatively, and at the 8-week follow-up examination. Only complications that occurred in the immediate 8-week post-operative period were included.

### Surgical technique

The anesthesia protocol typically consisted of IV premedication with midazolam 0.1 mg/kg (Pharmsource, Brunswick, GA) and hydromorphone 0.1 mg/kg (Pharmsource, Brunswick, GA). General anesthesia was induced with propofol 4–6 mg/kg IV (Pharmsource, Brunswick, GA) and maintained by inhalation anesthesia with isoflurane and oxygen. Regional anesthesia was provided in all dogs via a lumbosacral epidural injection with preservative-free morphine 0.15 mg/kg (Pharmsource, Brunswick, GA). Cefazolin 22 mg/kg IV (Pharmsource, Brunswick, GA) or ampicillin/sulbactam sodium 22 mg/kg IV (Pharmsource, Brunswick, GA) was administered approximately 45 min before the first incision and perioperatively at 90-min intervals during general anesthesia.

The patients were positioned in dorsal recumbency with the affected limb hanging. The entire limb was aseptically scrubbed with chlorhexidine solution. Four quarter drapes were placed around the proximal thigh. From the metatarsals distally, the limb was covered with an impermeable dressing and then covered with VetRap (3M Animal Care Products, St. Paul, MN, USA). A large patient drape was then applied over the limb. A craniomedial parapatellar arthrotomy was performed. A Hohmann retractor was used to maximally distract the joint for better visualization. A blunt-tipped probe was used to palpate the menisci. If the meniscus was normal, it was left intact. If the medial meniscus was damaged, the torn portion was removed by partial segmental meniscectomy [6]. If the damage was peripheral with detachment of the caudal horn of the medial meniscus from the joint capsule, a medial meniscal release at the caudal meniscotibial attachment was performed [6].

A standard TPLO procedure as described by Slocum without the use of a jig was performed in all cases by the same board-certified surgeon [3]. The incision site was thoroughly lavaged after placement of the plate and screws. The fascia, muscle, and subcutaneous tissues were routinely closed. The skin was closed with either staples or non-absorbable sutures. Orthogonal radiographs were acquired immediately postoperatively to confirm correct implant placement and appropriate postoperative TPA.

### Postoperative care

Postoperative analgesia consisted of hydromorphone 0.1 mg/kg IV every 4 hours (Pharmsource, Brunswick, GA) or a constant rate infusion of fentanyl (Pharmsource, Brunswick, GA) as needed. The patients were then transitioned to oral medications when they had resumed eating. All patients were discharged home postoperatively on tramadol (4 mg/kg every 8 hours orally) (MWI Animal Health, Boise, ID) and either cefpodoxime (5 mg/kg every 24 hours orally) (Pharmsource, Brunswick, GA) or amoxicillin/clavulanate potassium (13.75 mg/kg every 12 hours orally) (Covetrus, Portland, ME) for 7 days. Non-steroidal anti-inflammatories were not prescribed due to surgeon preference. No postoperative bandage was placed. Postoperative exercise restriction consisted of 8 weeks of confinement to a kennel or a small room, with 10–15 min of controlled walking on a leash three times daily. After 8 weeks, there was a gradual return to normal activity over a 2-week period. Clinical assessment and an incision check and suture/staple removal were performed at 2 weeks postoperatively. Clinical and radiographic reassessments were performed at 8 weeks postoperatively.

### Statistical analysis

The numeric variables of age, weight, preoperative TPA, postoperative TPA, and surgical time were compared between dogs with no complications and dogs with complications by Independent t-test. The association of the animal sex, neutering status, plate size, closure method (suture or staple), post-operative antibiotic used (cefpodoxime or amoxicillin/clavulanic acid) and meniscal treatment (normal, meniscectomy, or release) were compared against the outcome of no complications or complications by Chi-Square analysis. Weight was compared between plate sizes by ANOVA with a posthoc Newman-Keuls multiple comparisons test. The linear relationship between the numeric variables of age, weight, surgical time, preoperative TPA, and postoperative TPA were determined by Pearson’s correlation coefficient. The numeric variables of age, weight, preoperative TPA, postoperative TPA, and surgical time were tested for normality by Anderson-Darling. A p-value ≤0.05 was considered significant for all comparisons.

## Results

Ninety dogs weighing <15 kg met the inclusion criteria. Four (4.44%) of these dogs were intact males, 39 (43.33%) were neutered males, 2 (2.22%) were intact females, and 45 (50%) were spayed females. Ten dogs (11.11%) were mixed breed and 80 (88.88%) were purebred. The most common breeds were Yorkshire Terrier (n = 9), Havanese (n = 9), Maltese (n = 7), Bichon Frise (n = 6), and Cocker Spaniel (n = 6). Mean weight was 8.76 (range, 2.4–14.9) kg and mean age at the time of surgery was 7.63 (range, 1.83–15.66) years. Mean preoperative TPA was 29.45° (range, 23–37°) and mean postoperative TPA was 5.16° (range, 2–10°). Mean operating time was 75.1 (range, 45–120) minutes. Forty-five limbs had normal menisci, so no meniscal treatment was performed. Forty-five limbs had abnormal menisci; 13 underwent a meniscal release, and 32 underwent segmental meniscectomy. Forty-four 2.0-mm, 19 2.4-mm, and 27 2.7-mm DT locking Swiss style TPLO plates were placed (Veterinary Orthopedic Implants, St. Augustine, FL, USA). The TPLO procedures were performed on 90 stifles (left, n = 59; right, n = 31). The incisions were closed by either non-absorbable sutures (n = 48) or staples (n = 42). Postoperatively, 21 dogs received amoxicillin/clavulanate potassium and 69 received cefpodoxime. Radiographs performed at the 8-week post-operative examination revealed fully healed osteotomy sites. All dogs made a full recovery with resolution of lameness by the 8-week postoperative examination. Four dogs (4.44%) developed postoperative complications of partial incisional dehiscence, two of these cases had received amoxicillin/clavulanate potassium and two of these cases had received cefpodoxime in the postoperative period. None of these cases required surgical revision.

When the numeric variables of age, weight, preoperative TPA, postoperative TPA, and surgical time were compared between dogs with no complications and dogs with complications, there was no association present (Table 1). Chi-square analysis identified a significant association of a 2.4-mm plate size with complications (Table 2). The plate size used was significantly different by weight (Table 3). There were no statistically significant linear relationships between the numeric variables of age, weight, surgical time, preoperative TPA, and postoperative TPA by Pearson’s correlation coefficient (Table 4).

**Table 1:** Complication rates for selected variables.

Variable	No complications n = 86	Yes complications n = 4	p-value
Age, years	7.65 ± 2.88	7.31 ± 1.90	0.819
Weight, kg	8.78 ± 3.45	8.40 ± 2.36	0.838
Preoperative TPA, degrees	29.44 ± 2.05	29.75 ± 2.63	0.772
Postoperative TPA, degrees	5.15 ± 2.30	5.5 ± 1.29	0.765
Surgical time, minutes	74.69 ± 15.29	85.0 ± 26.46	0.205

TPA, tibial plateau angle

**Table 2:** Association of multiple variables with complications.

Variable	p-value
Plate size	0.024*
Closure	0.406
Meniscal treatment	0.553
Limb	0.139
Sex	0.265
Neutering status	0.585
Antibiotic administered	0.198

*Indicates that the 2.4mm plates were significantly more likely to have a complication.

**Table 3:** Dog weight versus plate size selected.

Plate size (mm)	Mean weight (kg)	n
2.0	6.14 ± 1.76^a^	44
2.4	9.08 ± 1.79^b^	19
2.7	12.81 ± 1.87^c^	27

Different subscript letters ^(a,b,c)^ indicate significant difference of weight between plate groups at p<0.05.

**Table 4:** Pearson’s correlation coefficients for numeric variables.

	Age	Weight	Surgical time	TPA
Age				
Weight	r = 0.0036 (p = 0.973)			
Surgical Time	r = -0.0688 (p = 0.519)	r = -0.0882 (p = 0.408)		
Preoperative TPA	r = 0.1672 (p = 0.115)	r = 0.0483 (p = 0.651)	r = -0.0972 (p = 0.362)	
Postoperative TPA	r = -0.017 (p = 0.877)	r = 0.143 (p = 0.180	r = 0.036 (p = 0.736)	r = 0.138 (p = 0.195)

## Discussion

To our knowledge, this is the largest case study to investigate the postoperative short-term complications of TPLO performed by a single surgeon for up to 8 weeks after surgery in dogs weighing <15 kg and to assess predictive variables. The overall complication rate was 4.44% (4/90 dogs). There were four cases of partial incisional dehiscence, none of which required surgical revision. Complications were significantly more likely in dogs that had undergone placement of a 2.4-mm TPLO plate. Dogs weighing <15 kg that underwent TPLO had good short-term outcomes with minimal complications. The overall complication rate after TPLO in dogs weighing <15 kg is less than that historically reported in heavier dogs. Our data suggests that TPLO is a safe treatment option in small dogs with cranial cruciate ligament rupture.

Complications reported following TPLO include fracture or avulsion of the tibial tuberosity, tibial or fibular fracture, MPL, subsequent meniscal tears, implant infection, implant failure, patellar tendonitis or desmitis, delayed union, and osteomyelitis [13, 14, 20]. In our study, there were four postoperative complications (4.44%) of partial incisional dehiscence that did not require a second surgical procedure. The four partial incisional dehiscence cases were allowed to heal by secondary intention and the incision sites healed completely. To our knowledge, there were no cases of subsequent implant infection.

In our study, there were 21 cases that received amoxicillin/clavulanate potassium post-operatively and 69 cases that received cefpodoxime post-operatively. It is possible that use of third generation cephalosporins may have resulted in fewer implant and incisional infections. At the time these operations were performed, Fitzpatrick et al suggested that postoperative antibiotics could protect against complications [13]. In a study reported in 2015, the prevalence of TPLO surgical site infection was 7.2% in patients that received postoperative antimicrobials and 15.0% in patients that did not. In that study, 6.0% of 150 cases that received cefpodoxime developed surgical site infections and 23.1% of 13 cases that received amoxicillin/clavulanate developed surgical site infections [15]. A study published in 2019 found that administration of antimicrobial medications after TPLO surgery did not protect against surgical site infections or predispose to antibiotic resistance [21]. The findings of our study indicate that even use of stronger antibiotics may not have any effect on outcomes. The surgeon’s rationale for use of cefpodoxime after TPLO surgery was its ease of administration; cefpodoxime is given every 24 hours whereas cephalexin is given every 8 hours.

It is also important to note the discrepancies in definitions of inflammation and infection when interpreting infection rates in the veterinary literature, which may be inaccurate as a result of failure to use standard definitions for surgical site infections and failure to differentiate infection from inflammation [22]. Post-TPLO infection rates of 0.8%-25.9% has been reported [8–11]. These rates are higher than those associated with other orthopedic procedures and even higher than those expected after clean surgical procedures (1.5%-2.6%) [23].

We examined if there was any correlation between TPLO complications and variables such as age, weight, preoperative TPA, postoperative TPA, and surgical time. As Table 1 shows, there was no association present with any of these variables. We also examined if there was any association between neuter status, closure, meniscal treatment, affected limb, sex, plate size and antibiotic administered. As Table 2 shows, there was no association present with any of these variables, except for the 2.4-mm TPLO plate size.

In this study, neuter status did not have any association with TPLO complications. The effect of neutering status may not have reached a statistical significance because of lack of adequate numbers of intact dogs in our study population to detect a difference since most patients seen by this referral hospital are neutered.

In this study, patients in which 2.4-mm TPLO plates were used were more likely to develop postoperative complications. Nineteen patients received a 2.4-mm TPLO plate and three of these cases developed partial incisional dehiscence as a complication. It is possible that the novelty of the 2.4mm locking TPLO plate at the time of the study, required a learning curve, resulting in an increased likelihood of complications.

Furthermore, as seen in Table 3, there was also a significant difference of the plate size used by weight of the patient as could be expected if dog size and weight are considered in the selection of plate size by the surgeon. All plate sizes were different from each other in terms of the mean dog weight they were placed on. This might explain why there were fewer postoperative complications in this study than those reported in previous studies.

Higher weight has been associated with an increased complication rate following TPLO [13, 20]. However, we did not find a correlation between weight and complication rates in dogs weighing <15 kg, likely due to the limitation of the upper limit of dog weight during the selection process. It is possible that a study limited to small-breed dogs but including dogs with a weight of >15 kg because of extreme obesity, would reach a different conclusion regarding weight-related complications.

Unlike in large-breed dogs, CCL pathology and its risk factors have not been extensively studied in small-breed dogs. Small dogs with CCL pathology have been reported to have a mean TPA of 27.4° (range, 21°-40°) [24–26]. A recent study that compared tibial conformation between normal Yorkshire Terriers and Labrador Retrievers found their mean TPA values to be 30° ± 4° and 25° ± 3°, respectively [27]. Given that our study was focused exclusively on dogs weighing <15 kg, we did not exclude dogs based on their TPA. In our study, the mean TPA was 29.45°. We did not find any complications associated with TPA. In a recent study of the long-term complications after TPLO in small dogs with a TPA >30°, minor complications were reported in 22.7% of cases [28]. Another study that evaluated the 8-week postoperative complication rate associated with TPLO using 2.0-mm and 2.7-mm plates reported an overall complication rate of 36% [29]. Fifty-two percent of the complications in that study were subclinical; when those cases were removed, the clinical complication rate was 17%. Our study differs in that we used three different plate sizes (2.0-mm, 2.4-mm and 2.7-mm) of locking TPLO plates and we focused solely on dogs weighing <15 kg. In another study that evaluated the outcomes of TPLO in small-breed dogs when 1.9-mm/2.5-mm conically-coupled locking plates were used, the overall complication rate was 15.2% [30]. Furthermore, Fitzpatrick et al reported a postoperative TPLO complication rate of 14.8% [13]. Our overall complication rate of 4.44% is less than that in previous TPLO studies, which included dogs weighing <15 kg and weighing >15 kg.

Dogs weighing <15 kg that underwent TPLO had good short-term outcomes with minimal complications. Our findings are consistent with those of a previous study in which objective gait analysis and clinical metrology confirmed an excellent outcome after TPLO in small dogs with CCL pathology [31]. A recent study reported in 2020, in which force plate gait analysis was performed after TPLO in small-breed dogs, found that the objective limb function in the affected hindlimb improved continuously after surgery and reached normal values at 6 months after surgery [32]. Our study yielded similar data in regard to short-term complications and had a good clinical outcome after TPLO in small dogs with a high TPA (>30°) [33, 34].

Small-breed dogs with concurrent MPL should also be considered. MPL is frequently documented in small-breed dogs and has often been reported to occur with rupture of the CCL [35–37]. It has been suggested that CCL rupture may occur later in life secondary to MPL in response to increased stress on the CCL [37]. It is possible that TPLO with simultaneous corrective MPL surgery may increase the risk of complications. However, dogs with concurrent MPL were excluded in our study, and a comparison of these techniques should be performed in the future.

This study has some limitations, including a retrospective design that relied on use of clinical records. Therefore, clinical outcome measures could not be evaluated. A kinematic gait analysis or owner assessment questionnaire would have been helpful for clinical evaluation of functional outcomes. Another limitation is that this study investigated TPLO procedures in dogs weighing <15kg up to only 8 weeks after surgery. The standard postoperative follow-up duration after TPLO surgery is variable; however, most surgeons only perform rechecks for up until 8 weeks postoperatively because this is the typical time taken for healing of the osteotomy. Typically, a gradual return to normal activity is recommended after the 8-week postoperative period. Since our study was retrospective in nature, assessment of clinical outcomes was based on physical examination and evaluation of radiographs by one surgeon only until 8 weeks postoperatively. All of the dogs in this study made a full recovery with resolution of lameness and healed osteotomies by the 8-week postoperative recheck examination. Furthermore, we identified complications by reviewing clinical records and cannot exclude the possibility that some complications were not documented or occurred after the 8-week postoperative period. The follow-up time may be considered short in this study; however, the goal was to assess short-term complications associated with TPLO in dogs weighing <15 kg. A longer follow-up, optimally for 1 year or longer, by performing a physical examination at 1-year post-surgery and/or contacting owners and primary veterinarians would have been helpful in an effort to obtain more data. This would also allow any latent implant infections or long-term complications that may have occurred.

Another limitation in our study is the difference of antibiotics used. Ideally, only perioperative antibiotics should have been used. Also, if postoperative antibiotic administration was used it should have been a first-generation cephalosporin such as cephalexin instead of a third-generation cephalosporin such as cefpodoxime in order to help prevent development of antibiotic resistance.

We also recognize that all of these cases were performed by a single board-certified surgeon, so there may have been fewer complications as a result of the experience of the surgeon performing TPLO in dogs weighing <15 kg. It is possible that residents or novice surgeons may have greater complication rates when performing TPLOs in dogs in this weight range.

In conclusion, the results of this study suggest that use of 2.4-mm TPLO plates was associated with an increased short-term postoperative complication rate in dogs weighing <15 kg. TPLO in dogs weighing <15 kg had good short-term outcomes with minimal complications. TPLO is well tolerated in small dogs with complication rates that are less than those historically reported for large dogs. The complications seen were minor and did not affect the 8-week postoperative outcomes. All of the dogs in this study made a full recovery with resolution of lameness and healed osteotomies by the 8-week postoperative recheck examination. Further studies, ideally with a prospective design and objective assessments, are needed to assess the long-term outcomes following single-sided TPLO, bilateral TPLO performed in the same session, concurrent correction of MPL, and complications after arthroscopy in dogs weighing <15 kg.
